# 5′CAG and 5′CTG Repeats Create Differential Impediment to the Progression of a Minimal Reconstituted T4 Replisome Depending on the Concentration of dNTPs

**DOI:** 10.4061/2011/213824

**Published:** 2011-08-10

**Authors:** Emmanuelle Delagoutte, Giuseppe Baldacci

**Affiliations:** ^1^Muséum National d'Histoire Naturelle, Département “Régulations, Développement et Diversité Moléculaire”, Laboratoire de Régulations et Dynamique des Génomes, USM 0503—INSERM U 565—UMR 7196, Case Postale no 26, 57 rue Cuvier, 75231 Paris cedex 05, France; ^2^Institut Jacques Monod, UMR7592, “Pathologies de la réplication de l'ADN”, CNRS and Université Paris-Diderot, 15 Rue Hélène Brion, 75205 Paris Cedex 13,, France

## Abstract

Instability of repetitive sequences originates from strand misalignment during repair or replicative DNA synthesis. To investigate the activity of reconstituted T4 replisomes across trinucleotide repeats (TNRs) during leading strand DNA synthesis, we developed a method to build replication miniforks containing a TNR unit of defined sequence and length. Each minifork consists of three strands, primer, leading strand template, and lagging strand template with a 5′ single-stranded (ss) tail. Each strand is prepared independently, and the minifork is assembled by hybridization of the three strands. Using these miniforks and a minimal reconstituted T4 replisome, we show that during leading strand DNA synthesis, the dNTP concentration dictates which strand of the structure-forming 5′CAG/5′CTG repeat creates the strongest impediment to the minimal replication complex. We discuss this result in the light of the known fluctuation of dNTP concentration during the cell cycle and cell growth and the known concentration balance among individual dNTPs.

## 1. Introduction

Repetitive sequences, such as trinucleotide repeats (TNRs), are spread all over the genome and can be found in intergenic regions, 5′ regulatory regions, promoters, introns, or exons. Such sequences are particularly prone to mutate, and their rate of mutation can be several orders of magnitude higher than that of bulk DNA [1]. A change in the repeat number of a given repetitive sequence can influence gene expression [2–4], allowing morphological evolution [5] or generation of diverse social behaviors [6]. On the other hand, a dozen genetic diseases (e.g., myotonic dystrophy, Huntington's disease, and a variety of ataxias) are caused by expansion of a TNR sequence located at a specific locus of the genome [7–11]. Similarly, a variety of cancers are due to frameshift mutations in the repetitive sequence of a given gene [12, 13]. The molecular mechanism underlying repetitive sequence instability has not been completely unraveled, and current models are based on strand slippage during replicative or repair DNA synthesis. Strand slippage is made possible by the repetitive nature of the sequence and leads to the formation of a hairpin on the template or newly synthesized strand. A recent study has provided direct evidence for hairpin formation during TNR replication *in vivo* [14] although the precise time at which hairpin forms is still unknown. Many of the *in vitro* studies published so far in the field of TNR instability relied on primer extension assays combining TNRs with different sequences and lengths and various DNA polymerases. If primer extension assays are good models of gap repair DNA synthesis, they are not fully adapted to study replicative DNA synthesis. The use of replication miniforks containing a defined TNR sequence on which a reconstituted replisome of various complexities can assemble should be very helpful in dissecting the mechanism involved in TNR instability during DNA replication. For instance, the activity of each component of the replisome can be easily examined and the experimental conditions changed without difficulty. The T4 replication machinery is an ideal system, since it is the simplest replication system that uses essentially the same set of regulatory proteins also used by higher organisms (for reviews, see [15–20]). It requires only seven proteins to initiate and catalyze coordinated *in vitro* leading and lagging strand DNA synthesis with a speed, processivity, and fidelity similar to those measured *in vivo*. Among the T4 replisomal proteins are (1) the DNA polymerase, the gene product (gp) 43, with a 5′→3′ DNA synthesis activity and a 3′→5′ exonuclease or proofreading activity that removes misincorporated dNMPs, (2) the helicase, gp41, with a 5′→3′ double-stranded (ds) DNA strand separation activity that unwinds the parental duplex by sterically excluding the ss leading strand template and encircling the ss lagging strand template, (3) the processivity factor, gp45, a homotrimeric ring-shaped and noncatalytic protein that confers processivity to the DNA polymerase, (4) the clamp loader, gp44/62, a hetero-oligomeric complex that loads the processivity factor at a primer-template (p-t) junction, (5) the single-stranded (ss) DNA binding protein (SSB), gp32, that binds and protects the naked ss DNA exposed by the helicase, and finally (6) the primase, gp61, that synthesizes small RNA primers complementary to the ss lagging strand template that the lagging strand DNA polymerase extends. An eighth T4-encoded protein called the helicase loader protein, gp59, is required to load the helicase on ss DNA covered by SSB. 

In this paper, we present a method to build replication miniforks containing a TNR of defined sequence and length. Each minifork consists of three strands, namely, primer, leading, and lagging strand template. The primer anneals to the 3′ end of the leading strand template to create a specific binding site, a p-t junction, for the DNA polymerase. The lagging strand template is complementary to the leading strand template except in its 5′ extremity. The ss tail at the 5′ end of the lagging strand template constitutes the assembly site of the replicative helicase. The method relies on the preparation of each strand of the minifork separately followed by the hybridization of the three strands of the minifork. The DNA synthesis activity of simple reconstituted bacteriophage T4 replisomes across specific ds TNR sequences was characterized. We show that a minimal replisome constituted by the T4 helicase (gp41) and the T4 DNA polymerase (gp43) can replicate the leading strand template of random, structure-forming or nonstructure-forming TNR sequences. The T4 helicase loader (gp59) increases the efficiency of strand displacement DNA synthesis of the minimal homologous couple and may reduce slippage of the helicase that is, hydrolysis of ATP nonassociated with ds DNA unwinding. In contrast, a heterologous couple constituted by the T4 helicase and Klenow fragment undergoes uncoordinated leading strand DNA synthesis, even in the presence of the T4 helicase loader. Coupled DNA synthesis at various concentrations of dNTPs was performed and surprisingly revealed that the strand of the structure-forming 5′CAG/5′CTG TNR that hinders the progression of the reconstituted T4 replication trio (gp41-gp43-gp59) depends on the concentration of dNTPs. For instance, at high [dNTPs], the greatest impediment to progression of the T4 replication trio is measured when the 5′CAG sequence is on the leading strand template. In contrast, at low [dNTPs], the 5′CTG sequence on the leading strand template creates a greater impediment to T4 replication trio progression than the 5′CAG sequence due to a very low efficiency of incorporation of dAMP across TMP of the 5′CTG repeat. The functional consequences of these results are discussed.

## 2. Materials and Methods

### 2.1. Enzymes

Herculase-enhanced DNA polymerase was from Stratagene; Phusion high-fidelity DNA polymerase was from Finnzymes; T7 exonuclease, T4 polynucleotide kinase (PNK), and Klenow fragment were from New England Biolabs (NEB). T4 helicase (gp41), T4 DNA polymerase (gp43), and T4 helicase loader (gp59) were prepared as described [21–25]. The proteins purified in our laboratory were estimated to be at least 90% pure by Coomassie Blue staining. Their concentration was measured by UV spectroscopy using an extinction coefficient of 7.6 × 10^4^, 1.3 × 10^5^, 3.8 × 10^4^ M^−1^cm^−1^ for monomeric gp41, monomeric gp43 and monomeric gp59, respectively.

### 2.2. DNAs

The three plasmids derived from pcDNA3 (Invitrogen) and containing a TNR of defined length and sequence are listed and described in Table 1. The plasmid p-Empty also derives from pcDNA3 but contains no repetitive sequence. Plasmid constructions are named based on the sequence of the leading template strand. Oligonucleotides were from Eurogentec and their sequence is listed in Table 1 as is also their annealing position with respect to the repetitive sequence. The oligonucleotides (50T/4ps)/p867 and 1033/4ps carry four phosphorothioate linkages at their 5′ extremity that make them resistant to the 5′→3′ T7 exonuclease. All oligonucleotides were gel-purified before use. Their concentrations were determined by UV spectroscopy using the extinction coefficients provided by the manufacturer.

### 2.3. PCR Conditions

Polymerase chain reactions (PCRs) were performed in the buffer of the DNA polymerase provided by the manufacturer. The concentrations of plasmid, primers, dNTPs, and enzyme were as recommended by the manufacturer, except for the oligonucleotide that contains the phosphorothioate linkages whose concentration is half the concentration recommended by the manufacturer. The cycling conditions when using the Phusion high-fidelity DNA polymerase were as follows: initial denaturation (3 min at 98°C); 25 cycles of the three following steps (10 sec at 98°C, 10 sec at 63°C, 15 sec at 72°C); final extension (10 min at 72°C). The cycling conditions when using the Herculase-enhanced DNA polymerase were as follows: initial denaturation (3 min at 98°C), and 25 cycles of the three following steps (40 sec at 98°C, 30 sec at 60°C, and 30 sec at 72°C), and final extension (10 min at 72°C). After PCR, the Nucleospin extract kit (Macherey-Nagel) was used to remove unused primers as recommended by the manufacturer. The PCR products were analyzed by electrophoresis on a 2% agarose gel in 1X TBE.

### 2.4. T7 Exonuclease Digestion

T7 exonuclease digestion was performed in 1X NEB buffer 4 (50 mM KOAc, 20 mM Tris-OAc, 10 mM MgOAc_2_ and 1 mM DTT pH 7.9 at 25°C) at 25°C. Prior to large scale digestion, the enzyme concentration and the duration of the digestion were adjusted by performing a small-scale digestion. The large-scale T7 exonuclease digestion was stopped by adding EDTA (final concentration of 80 mM) and proteinase K (final concentration of 1.5 mg/mL), and incubated for 10 min at 37°C and 10 min at 65°C. T7 digestion was checked by electrophoresis on a 2% agarose gel in 1X TBE.

### 2.5. Radiolabelling of p821

Radiolabelling of the oligonucleotide p821 at its 5′ extremity was performed by incubating 1 *μ*M oligonucleotide with 1 *μ*M {*γ* ^32^P}-ATP, 1 *μ*M ATP and PNK (0.2 u/mL) in 1X PNK buffer (70 mM Tris-HCl, 10 mM MgCl_2_ and 5 mM DTT pH 7.6 at 25°C) for 45 min at 37°C. Nonincorporated {*γ* ^32^P}-ATP and ATP were removed using a Biospin 6 column (Biorad) equilibrated in TE buffer.

### 2.6. Preparation of the Minifork

Miniforks were prepared by mixing the radiolabelled p821 primer with the ss leading and lagging strand templates in a buffer containing 40 mM Tris-HCl pH 7.5, 20 mM MgCl_2_, and 50 mM NaCl. Strand hybridization was performed by heating (5 min at 95°C) and slow cooling. Hybridization was checked by electrophoresis on a native polyacrylamide gel. The mass ratio of acrylamide to bisacrylamide was 29 : 1 and native gels were in 1X TBE.

### 2.7. DNA Synthesis Assay

Except for Klenow fragment, protein concentrations are given in monomeric units. DNA synthesis was performed in the buffer used to prepare the miniforks supplemented with 250 *μ*M each dNTP (unless indicated otherwise), 2.5 mM ATP, and 2.5 mM DTT. The DNA, gp43, gp41 and gp59 concentrations were 10 nM, 30 nM, 200 nM, and 200 nM, respectively. Klenow fragment was either at 1 mU/*μ*L or 10 mU/*μ*L as indicated in the figure. DNA and DNA polymerase (gp43 or Klenow fragment) were preincubated for 3 min at 37°C before adding gp41 premixed or not with gp59. At the indicated times, the reaction was quenched by adding EDTA to 50 mM. Proteinase K and SDS were added to a final concentration of 3 mg/mL and 0.05%, respectively, and proteolysis performed for 20 min at 37°C. One volume of denaturing blue (100% formamide, 0.01% bromophenol blue, and 0.01% xylene cyanol) was added to the samples that were then heated for 5 min at 95°C and loaded onto a 10% acrylamide sequencing gel (mass ratio of acrylamide to bisacrylamide = 19 : 1) in 1X TBE. After electrophoresis, the gel was dried and exposed on a phosphorimager screen. After at least 10 hours of exposure, the screen was scanned with a Storm 820 (GE Healthcare). The samples in the gel were quantified using ImageQuant version 5.1 or NT.

### 2.8. DNA Sequencing of the Leading Strand Template

The DNA substrates used for sequencing the ss leading strand templates were p-t junctions prepared in the 1X Sequenase Version 2.0 reaction buffer (40 mM Tris-HCl pH 7.5, 20 mM MgCl_2_ and 50 mM NaCl) and consisted of the radiolabelled p821 annealed to the ss leading strand template. The p-t junctions were treated for sequencing as recommended in the Sequenase Version 2.0 T7 DNA polymerase kit (Usb).

### 2.9. ATPase Assay

Assays were performed in the buffer used to prepare the miniforks supplemented with 250 *μ*M of each dNTP, 2.5 mM ATP, 0.1 *μ*M {*γ* ^32^P}-ATP and 2.5 mM DTT. The DNA, gp43, gp41 and gp59 concentrations were 10 nM, 30 nM, 200 nM and 200 nM, respectively (concentrations expressed in monomeric units). When present, gp43 was preincubated with the minifork for 3 min at 37°C before adding gp41 premixed or not with gp59. At the indicated times the reaction was quenched by spotting an aliquot of the reaction on a PEI cellulose thin layer chromatography (TLC) plate. ATP and inorganic phosphate were separated by running the TLC plate in 0.35 M potassium phosphate buffer (pH 3). The TLC plate was next air-dried and exposed on a phosphorimager screen. After at least 10 hours of exposure, the screen was scanned with a Storm 820 (GE Healthcare). Radioactive ATP and inorganic radioactive phosphate were quantified using ImageQuant version 5.1 or NT.

## 3. Results

### 3.1. Strategy

**Figure 1:**
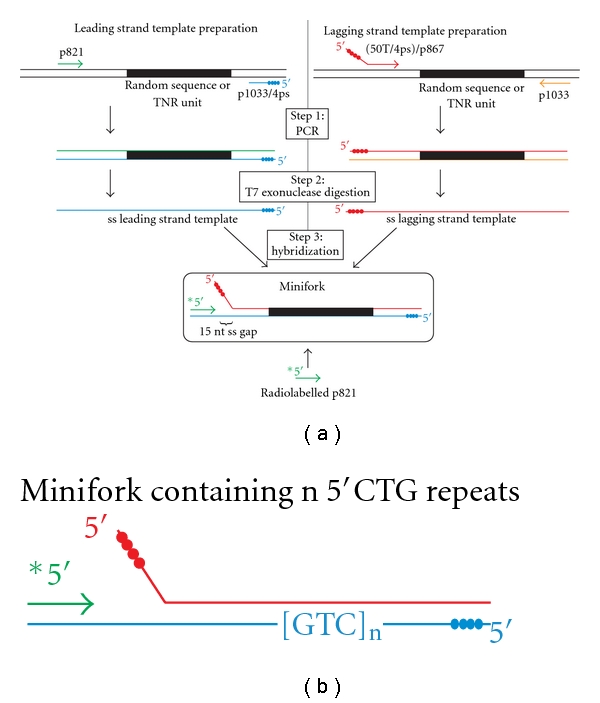
Strategy of the preparation of model replication miniforks. (a) The left and right sides of the figure correspond to the strategy used to build the ss leading and lagging strand templates of the minifork, respectively. The ss leading and lagging strand templates are combined with the radiolabelled p821 primer to assemble the minifork by strand hybridization. Replication miniforks are prepared in three consecutive steps. The first step (Step 1: PCR) consists of a PCR using plasmids containing a random or a TNR sequence (shown as a black rectangle) and oligonucleotides that flank the random sequence or the TNR unit. For each PCR, one of the oligonucleotides (p1033/4ps (colored in blue) for the preparation of the ss leading strand template, and (50T/4ps)/p867 (colored in red) for the preparation of the ss lagging strand template) carries 4 phosphorothioate linkages (represented as filled blue and red spheres for p1033/4ps and (50T/4ps)/p867, resp.) at its 5′ end. After PCR, the ds PCR products are digested by the T7 exonuclease that specifically degrades the DNA strand (colored in green or orange for the preparation of the ss leading or lagging strand template, resp.) that does not contain the phosphorothioate linkages (Step 2: T7 exonuclease digestion). The minifork (shown in a rounded rectangle) is assembled by hybridization of the ss leading and lagging strand templates and the radiolabelled p821 primer (in green) (Step 3: Hybridization). A gap of 15 nts exists between the 3′ end of the p821 primer and the base of the ss tail of the lagging strand template to facilitate the assembly of the DNA polymerase at the p-t junction. (b) A minifork containing n repeats of 5′CTG is shown. The repeats are located on the leading strand template.

The strategy used to build replication miniforks of defined sequences is shown in Figure 1(a) and can be divided into three steps. The ss leading and lagging strand templates are prepared separately by a two-step procedure. In a first step, a PCR is performed with a plasmid containing a random or a TNR sequence (Table 1) and two oligonucleotides, one of them carrying a track of four phosphorothioate linkages at its 5′ extremity to make it resistant to the 5′→3′ T7 exonuclease. The oligonucleotide couples for the leading and lagging strand templates are (p821, p1033/4ps) and ((50T/4ps)/p867, p1033), respectively (see Table 1 for oligonucleotide sequences and annealing positions). In a second step, the PCR fragments are treated with T7 exonuclease to specifically degrade the strand that is devoid of phosphorothioate linkage. Miniforks are assembled in a third step by annealing the radiolabelled p821 primer, the ss leading and the lagging strand templates by a heating and slow cooling procedure. Each minifork (shown in a rounded rectangle in Figure 1(a)) carries a p-t junction on which the DNA polymerase can bind to initiate DNA synthesis and a 5′ ss tail on which the helicase with or without the assistance of the helicase loader can assemble. The 15 nucleotide (nt) gap of ss DNA that exists between the 3′ end of the p821 primer and the base of the ss tail of the lagging strand template (Figure 1(a)) makes it possible for the DNA polymerase to assemble and initiate DNA synthesis by filling the 15 nt-long gap of ss DNA before starting coupled DNA synthesis with the helicase. In what follows, the name of the TNR associated with a minifork refers to the TNR sequence of the leading strand template. For instance, the minifork containing a 5′CTG repeat has the 5′CTG repeat unit located on its leading strand template (Figure 1(b)).

### 3.2. Preparation of Leading and Lagging Templates of the Miniforks

**Figure 2:**
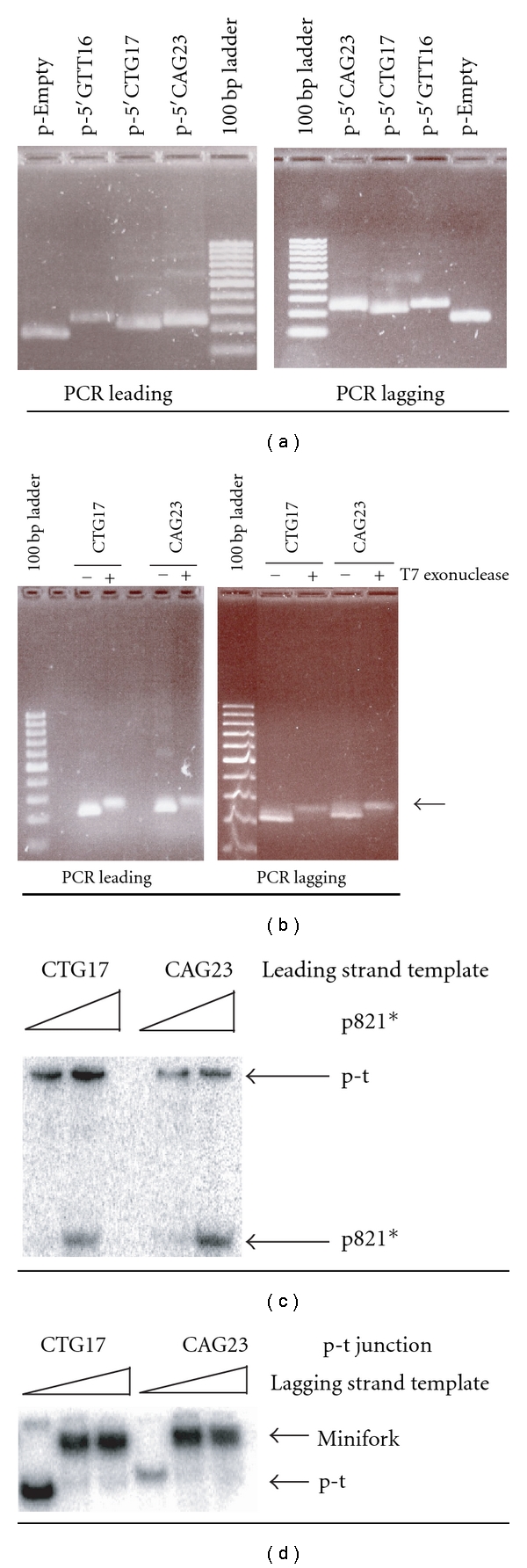
Preparation of leading and lagging strand templates, p-t junctions and miniforks. The DNA fragments in the 100 base pair (bp) ladder are 100, 200, 300, 400, 500/517, 600, 700, 800, 900, and 1000 pbs. (a) The agarose gels stained by ethidium bromide (EtBr) show the products of the PCR obtained with the four plasmids (p-Empty, p-5′GTT16, p-5′CTG17, and p-5′CAG23) and the oligonucleotide couple specific of the leading (“PCR leading”; left) or the lagging (“PCR lagging”; right) strand. The name of the plasmids used for the PCR is indicated at the top of the gels. (b) The agarose gels stained by EtBr show the products of the T7 exonuclease digestion. The PCR products obtained with p-5′CTG17 (CTG17) and p-5′CAG23 (CAG23) and the oligonucleotide couple specific of the leading (“PCR leading”; left) or the lagging (“PCR lagging”; right) strand were treated (+) or not (−) by T7 exonuclease. After treatment with T7 exonuclease, the appearance of a DNA band with a slower electrophoretic migration and a weaker intensity (indicated by a backward arrow) than the ds DNA is indicative of ss DNA production. (c) The ss leading strand templates containing either 17 repeats of CTG (CTG17) or 23 repeats of (CAG23) are mixed with increasing amounts of radiolabelled p821 (p821*) to generate the p-t junctions. Species are resolved on a native gel. Free p821 migrates faster than the p-t junctions. (d) The p-t junctions containing 17 repeats of CTG (CTG17) or 23 repeats of (CAG23) on their leading strand are mixed with increasing amounts of ss lagging strand template to assemble the miniforks. Species are resolved on a native gel. The miniforks migrate more slowly than the p-t junctions.

Two different DNA polymerases were tested, the Herculase-enhanced DNA polymerase and the Phusion high-fidelity DNA polymerase, and both gave suitable results. To use all the oligonucleotide that carried the phosphorothioate linkages, its concentration was half the concentration recommended by the manufacturer. Each plasmid (p-Empty, p-5′GTT16, p-5′CTG17, and p-5′CAG23; Table 1) was used with the oligonucleotide couple specific of the leading or lagging strand template. A PCR fragment of expected size was synthesized in each case (Figure 2(a)). The PCR fragments were next treated with T7 exonuclease to generate ss DNA. As shown in Figure 2(b) (data shown for the production of the ss leading templates containing 17 repeats of CTG and 23 repeats of CAG (left panel) and the ss lagging strand templates containing 17 repeats of CAG and 23 repeats of CTG (right panel)), a DNA band with a slower mobility and a weaker intensity than the untreated ds PCR fragment appeared after T7 exonuclease treatment, indicative of the production of ss DNA.

### 3.3. Assembly of the Miniforks

All miniforks were rendered radioactive by the use of the radiolabelled p821 oligonucleotide. A two-step procedure was followed to assemble the miniforks. First, radiolabelled p821 was annealed to the ss leading strand template to generate a p-t junction. Hybridization of the two DNAs was checked by electrophoresis on a native gel, because the free p821 primer and the p-t junction do not have the same electrophoretic mobility. Free p821 indeed migrated faster than the p-t junctions (Figure 2(c)). Second, the ss lagging strand template was annealed to the p-t junction, and strand hybridization similarly checked by electrophoresis on a native gel (Figure 2(d)).

### 3.4. Coupled DNA Synthesis across the ds Random Sequence of the Minifork

**Figure 3:**
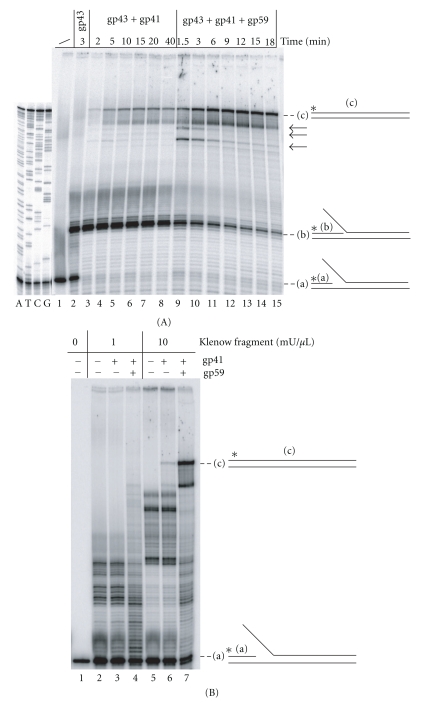
DNA synthesis activity of minimal reconstituted T4 replisomes across a minifork containing a random sequence. The minifork was prepared with the plasmid p-Empty and thus contains a random sequence to be replicated. gp43, gp41, and gp59 are the DNA polymerase, the helicase and the helicase loader of bacteriophage T4, respectively. (A) Gp43 was incubated with the minifork, alone (lane 2), with gp41 (lanes 3–8) or with gp41 and gp59 (lanes 9–15). The reaction was quenched at various times and the samples were loaded on a denaturing sequencing gel. (a) corresponds to the radiolabelled p821 primer. (b) corresponds to radiolabelled p821 extended by 15 nts up to the base of the 5′ ss tail of the lagging strand template. (c) corresponds to the radiolabelled p821 extended up to the end of the leading strand template after strand displacement DNA synthesis. The (a), (b), and (c) DNAs are also shown in the context of the minifork on the right side of the figure. Major transient intermediate products are indicated by backward arrows. The four sequencing reactions (A, T, C, and G) of the leading strand template of the minifork are shown on the left side of the figure. (B) The minifork was incubated with Klenow fragment alone (lanes 2 and 5), together with gp41 (lanes 3 and 6) or with gp41 and gp59 (lanes 4 and 7) for 20 minutes. The reaction products were resolved on a denaturing sequencing gel. Two amounts of Klenow fragment were tested (1 mU/*μ*L, lanes 2–4; 10 mU/*μ*L, lanes 5–7). (a) and (c) are as in 3A.

To test the quality of the miniforks, we first characterized the activity of the T4 DNA polymerase (gp43) alone or assisted by the T4 helicase (gp41) and T4 helicase loader (gp59) on a minifork carrying a random sequence. Gp43 was incubated for 3 min with the minifork before adding gp41 premixed or not with gp59 to initiate strand displacement DNA synthesis. As expected, during the first 3 min, gp43 filled the ss gap of the minifork up to the base of the ss lagging tail (Figure 3(A), lane 2). Gp43 was unable to efficiently synthesize through the DNA duplex due to a very weak intrinsic strand displacement activity, and a DNA intermediate corresponding to the p821 primer extended by 15 nts (labelled (b) in Figure 3(A)) accumulated. Addition of gp41 allowed coupled DNA synthesis across the duplex part of the minifork to take place (Figure 3(A), lanes 3–8) as pointed out by the accumulation of the full-length product (labelled (c) in Figure 3(A)) over time. Coupled leading strand DNA synthesis was highly stimulated by gp59 as a significantly higher amount of full length product accumulated over time when gp59 was part of the reconstituted replisome (Figure 3(A), lanes 9–15). In addition, in the presence of gp43, gp41, and gp59, three intermediate DNA synthesis products (indicated by a backward arrow in Figure 3(A)) near the end of the parental DNA duplex transiently accumulated to a significant extent; they might correspond to pause sites for the minimal reconstituted replisome from where DNA synthesis successfully resumed. 

Similarly, the DNA synthesis pattern was examined in the presence of Klenow fragment. Klenow fragment has weak strand displacement DNA synthesis activity that allowed it to synthesize through the duplex part of the minifork (Figure 3(B), lanes 2 and 5). However, contrary to the gp43-gp41 replication couple, DNA synthesis performed by Klenow fragment was not stimulated by gp41 (Figure 3(B), compare lanes 2, 3, 5, and 6). Under both conditions (with or without gp41), DNA synthesis was highly distributive as indicated by the numerous pause sites that were clearly visible along the template strand. The addition of gp59 to the Klenow fragment-gp41 replication couple stimulated DNA synthesis (Figure 3(B), compare lanes 3, 4, 6, and 7), but DNA synthesis remained distributive, suggesting uncoordinated DNA synthesis with this heterologous replication system.

### 3.5. At High Concentrations of dNTPs, a 5′CAG Repeat Creates a Greater Impediment to the T4 gp41-gp43-gp59 Replication Trio than a 5′CTG Repeat

**Figure 4:**
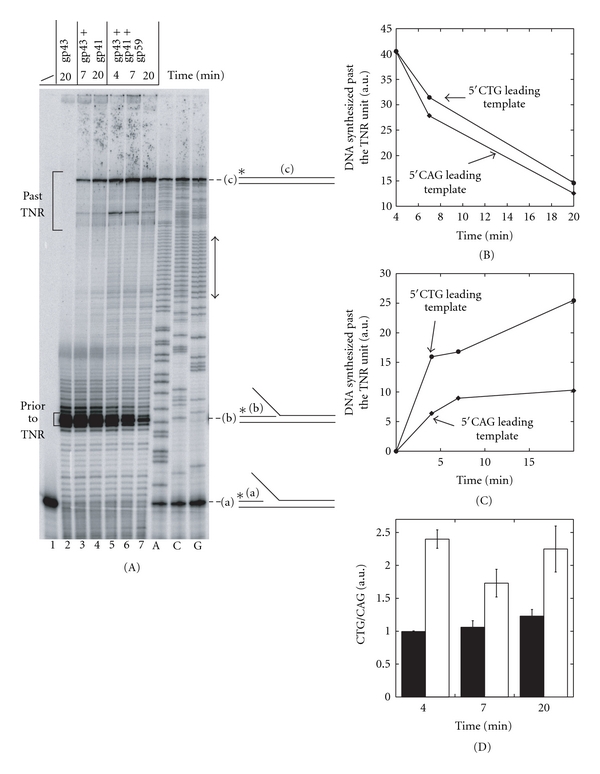
At high concentrations of dNTPs, 5′CAG repeats create a greater impediment than 5′CTG repeats to the gp41-gp43-gp59 replication trio. (A) The leading strand template of the minifork carries 17 repeats of 5′CTG. Three of the four sequencing reactions (A, C, G) of the leading strand template of the minifork are shown on the right side of the figure. The TNR unit is designated by a double-headed arrow. The minifork was incubated with gp43 alone (lane 2), with gp43 and gp41 (lanes 3 and 4), or with gp43, gp41 and gp59 (lanes 5–7). The reaction was quenched at various times and the samples were loaded onto a denaturing sequencing gel. (a), (b), and (c) are as in Figure 3. The primers extended by 14 to 16 nts are shown in the bracket labelled “prior to TNR” and are used to estimate the rate of the gp41-gp43-gp59 replication trio before the TNR unit. The elongation products that contain the full-length TNR are shown in the bracket labelled “past TNR” and are used to estimate the rate of the minimal reconstituted T4 replisome across the TNR unit. (B) The graph shows the decrease of intensity of primers elongated by 14 to 16 nts (indicated by the bracket labelled “prior to TNR” in 4) as a function of time for the miniforks containing 5′CTG or 5′CAG repeats on their leading strand template. (C) The graph shows the accumulation of DNA synthesis products that contain the full-length TNR unit (indicated as a bracket labelled “past TNR” in A) as a function of time for the miniforks containing 5′CTG or 5′CAG repeats on their leading strand template. (D) Black bars: ratio between the amount of primers extended by 14 to 16 nts measured with miniforks containing 5′CTG repeats and the amount of primers extended by 14 to 16 nts measured with the miniforks containing 5′CAG repeats at different times. White bars: ratio between the amount of DNA synthesis products that contain the full-length CAG unit and the amount of DNA synthesis products that contain the full-length CTG unit at different times. The error bars correspond to the standard deviation calculated from at least two independent experiments. B and C were obtained from quantifying the experiment presented in 4(A), and the experiment was performed in parallel with a minifork containing 23 5′CAG repeats. a.u.: arbitrary unit.

**Figure 5:**
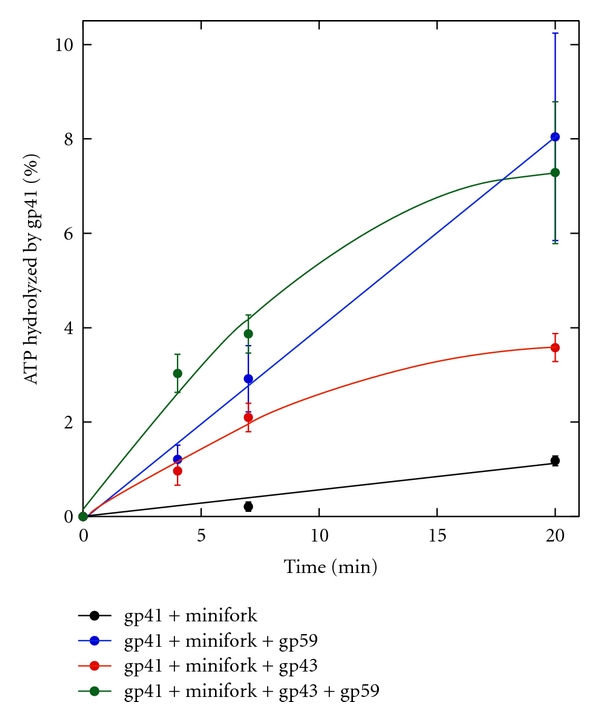
% ATP hydrolyzed by gp41 as a function of time in the presence of the minifork containing 17 repeats of 5′CTG and various proteins. The leading strand template of the minifork carried 17 repeats of 5′CTG and was included in all assays. The gp41 ATPase activity was measured as a function of time under various conditions: black filled circles and black curve (gp41 + minifork); blue filled circles and blue curve (gp41 + minifork + gp59); red filled circles and red curve (gp41 + minifork + gp43); green filled circles and green curve (gp41 + minifork + gp43 + gp59).

Miniforks containing 16 repeats of 5′GTT, 17 repeats of 5′CTG, or 23 repeats of 5′CAG on their leading strand template were built and the activity of minimal reconstituted T4 replisomes was tested on these miniforks. Similarly to the minifork containing a random sequence, the gp41-gp43 replication couple synthesized DNA quite efficiently across a nonstructure-forming (e.g., 5′GTT) or structure-forming TNRs (e.g., 5′CAG and 5′CTG) (Figure 4(A), result shown only for the minifork containing 17 repeats of 5′CTG, lanes 3 and 4). Addition of gp59 stimulated leading strand DNA synthesis fivefold (Figure 4(A), compare lanes 3, 6, 4, and 7). A similar stimulation of DNA synthesis by gp59, was measured with miniforks containing 16 repeats of 5′GTT or 23 repeats of 5′CAG (data not shown). The effect of gp59 on the ATPase activity of gp41 during coupled leading strand DNA synthesis was also evaluated. The gp41 ATPase activity during coupled leading strand DNA synthesis was measured in the presence or absence of gp59 and was compared to the activity measured in the absence of gp43 (Figure 5, result shown only for the minifork containing 17 repeats of 5′CTG). As expected [24], in the presence of the minifork as nucleic acid cofactor, gp59 stimulated the gp41 ATPase activity in the absence of gp43 (Figure 5, compare black and blue curves). This stimulation level is higher than reported in the absence of ss DNA (≤2, [24]), indicating that under our experimental conditions, gp59 stimulates both the intrinsic and the ss DNA-dependent ATPase activity of gp41. Gp43 by itself was also able to stimulate the gp41 ATPase activity (Figure 5, compare black and red curves), but to a slightly lower extent than gp59 (Figure 5, compare red and blue curves). Nevertheless, the addition of gp59 to the gp41-gp43 replication couple led to a gp41 ATPase activity comparable to the one measured for the gp41-gp59 couple (Figure 5, compare green and blue curves). A similar trend of stimulation of the gp41 ATPase activity by gp59 was measured with miniforks containing 16 repeats of 5′GTT or 23 repeats of 5′CAG (data not shown). The fact that gp59 stimulates the DNA synthesis activity of the gp41-gp43 replication couple to a higher extent than it does the ATPase activity (fivefold (Figure 4(A), compare lanes 3, 6, 4, and 7) *versus* twofold (Figure 5, compare red and green curves)) possibly reflects a reduced amount of slippage of gp41 in the presence of gp59 during coupled strand displacement DNA synthesis. 

DNA synthesis activity of the reconstituted gp41-gp43-gp59 replication trio across the 5′CAG and 5′CTG repeat unit was quantified. The rates of the minimal T4 replisome prior to, and across the TNR unit were estimated using the method previously described [26]. Briefly, the speed of the minimal reconstituted replisome before the TNR was determined by estimating the loss of signal intensity of primers extended by 14 to 16 nts (indicated by the bracket labelled “prior to TNR” in Figure 4(A)). The intensity of primers of these lengths decreased with time, since they were intermediate DNA synthesis products of larger extension products. To assess the effects of passage through the TNR region, the amount of elongated primers after passage of the minimal reconstituted replisome was measured. The amount of elongation products that contained the full length TNR unit (indicated by the bracket labelled “past TNR”) increased over time, since these products accumulated as coupled leading strand DNA synthesis proceeded. The results indicated that before reaching the TNR unit, the reconstituted gp41-gp43-gp59 replication trio assembled on the minifork containing a 5′CAG unit synthesized DNA at a speed very similar to that of the reconstituted gp41-gp43-gp59 replication trio assembled on the minifork containing a 5′CTG unit (Figures 4(B) and 4(D)). However, replicating through a 5′CAG repeat created a greater impediment to the minimal T4 replisome than replicating through a 5′CTG sequence. The amount of synthesized DNA containing the full-length TNR unit was indeed lower when the gp41-gp43-gp59 replication complex replicated through a 5′CAG template than when it replicated through the 5′CTG repeat unit (Figures 4(C) and 4(D)). This result is in a perfect agreement with the relative degree of impediment created by 5′CAG and 5′CTG repeats when these TNR sequences are presented to gp43 in a ss context [26].

### 3.6. At Low Concentrations of dNTPs, a 5′CAG Repeat Creates a Weaker Impediment to the Minimal Reconstituted T4 Replisome than a 5′CTG Repeat

**Figure 6:**
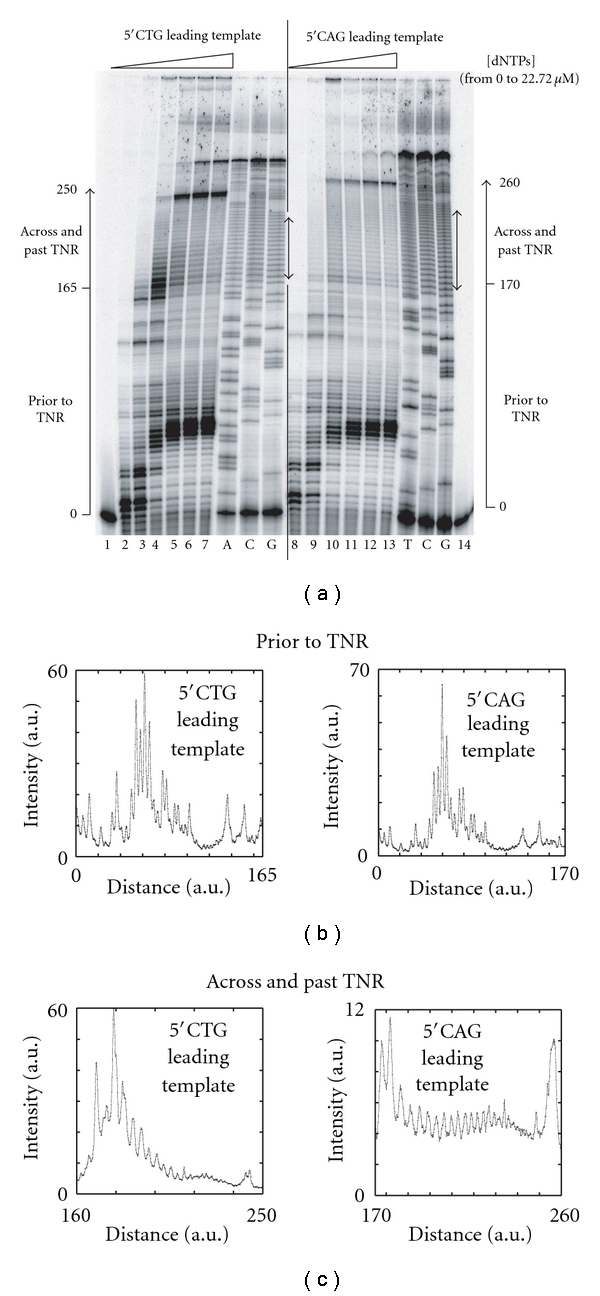
At low concentrations of dNTPs, 5′CTG repeats create a greater impediment than 5′CAG repeats to the gp41-gp43-gp59 replication trio. (a) Miniforks containing 17 5′CTG repeats (left panel) and 23 5′CAG repeats (right panel) on the leading strand template were incubated with gp43, gp41, and gp59 for 10 min at various concentrations of dNTPs, and the reaction products were resolved on a denaturing sequencing gel. Each of the four dNTPs is present at the following concentration (in *μ*M): 0 (lanes 1 and 14); 0.71 (lanes 2 and 8); 1.42 (lanes 3 and 9); 2.84 (lanes 4 and 10); 5.68 (lanes 5 and 11); 11.36 (lanes 6 and 12); 22.72 (lanes 7 and 13). Three of the four sequencing reactions (A, C, G for the minifork containing the 5′CTG repeats, left; T, C, G for the minifork containing the 5′CAG repeats, right) of the leading strand template of the minifork are shown on the right side of the dNTP titrations. The TNR unit is designated by a double-headed arrow. The lines along which DNA synthesis profiles at 2.84 *μ*M dNTPs (lanes 4 and 10) have been established are shown on the side of each panel and are divided into two parts labelled “prior to TNR” (from position 0 to 165 for the miniforks containing the 5′CTG repeats or position 0 to 170 for the miniforks containing the 5′CAG repeats) and “across and past TNR” (from position 160 to 250 for the miniforks containing the 5′CTG repeats or position 170 to 260 for the miniforks containing the 5′CAG repeats). (b) DNA synthesis profile at 2.84 *μ*M dNTPs prior to the TNR unit from position 0 to 165 for the miniforks containing the 5′CTG repeat (left panel) and from position 0 to 170 for the miniforks containing the 5′CAG repeats (right panel). (c) DNA synthesis profile at 2.84 *μ*M dNTPs across and beyond the TNR unit from position 160 to 250 for the miniforks containing the 5′CTG repeat (left panel) and from position 170 to 260 for the miniforks containing the 5′CAG repeats (right panel). a.u.: arbitrary unit.

At high dNTP concentrations, the reconstituted gp41-gp43-gp59 replication trio copied quite efficiently a leading strand template containing random, structure-forming or nonstructure-forming TNR sequences, and full-length product accumulated over time. However, such experimental conditions did not provide the opportunity to characterize the activity of the minimal replisome across the TNR unit itself. Therefore, the speed of the gp41-gp43-gp59 replication trio was reduced by lowering the concentrations of the dNTPs and the DNA synthesis pattern was examined under these conditions. As expected, reducing the concentrations of dNTPs decreased the size of the DNA products that were synthesized by the minimal reconstituted T4 replisome during leading strand DNA synthesis (Figure 6(a)). The DNA synthesis profile prior to, across, and beyond the TNR unit was established at 2.84 *μ*M dNTPs for the miniforks containing 5′CTG (Figure 6(a), lane 4) and 5′CAG (Figure 6(a), lane 10) repeats. The results showed that the DNA synthesis profiles prior to the TNR unit were very similar for both miniforks (Figure 6(b)) indicating that the sequence downstream of the TNR unit did not influence the activity of the reconstituted replication complex. In contrast, a strong blockage of DNA synthesis was observed as soon as the gp41-gp43-gp59 replication trio hit the 5′CTG sequence (Figure 6(c), left panel). This strong block was specific to the minifork containing the 5′CTG sequence as a smooth profile of DNA synthesis was observed with the minifork containing the 5′CAG sequence (Figure 6(c), right panel). These results showed that at low concentrations of dNTPs, the 5′CTG repeat created a greater impediment to the minimal reconstituted T4 replication complex than the 5′CAG repeat. Consequently, the situation at low concentrations of dNTPs differed from that observed at high concentrations of dNTPs, because at high concentrations of dNTPs, the 5′CAG repeat created a greater impediment to the minimal reconstituted T4 replication complex than the 5′CTG repeat (Figures 4(B)–4(D)). All together, our data suggest that the steps that control the DNA synthesis reaction at low and high concentrations of dNTPs are different.

### 3.7. Incorporation of dAMP Across TMP in the 5′CTG Sequence Context Is a More Difficult Reaction than the Incorporation of TMP across dAMP in the 5′CAG Sequence Context

**Figure 7:**
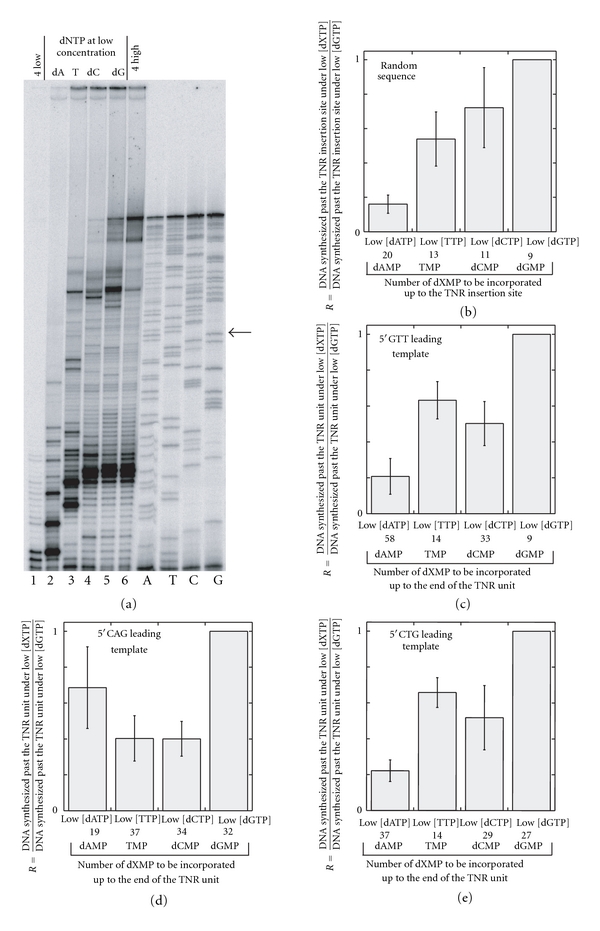
DNA synthesis profiles and efficiencies at various concentrations of dNTPs. (a) The minifork used here carries a random sequence. The four sequencing reactions (A, T, C, and G) of the leading strand template of the minifork are shown on the right side of the gel. The minifork was incubated with gp43, gp41 and gp59 for 10 min with the indicated dNTP mixture and the samples were loaded onto a denaturing sequencing gel. “4 low” (lane 1) means that the 4 dNTPs are at a low concentration (1 *μ*M). “4 high” (lane 6) means that the 4 dNTPs are at a high concentration (80 *μ*M). The single dNTP at low concentration (1 *μ*M) is indicated on the top of the figure (dATP, TTP, dCTP, and dGTP in lanes 2, 3, 4, and 5, resp.). The three other dNTPs are at 80 *μ*M. The backward arrow points to the TNR insertion site. (b) The gel shown in 7(a) has been quantified and the ratio R is shown for each reaction condition (low [dATP], low [TTP], low [dCTP], and low [dGTP]). R is the ratio between the amount of DNA synthesized past the TNR insertion site under a given condition of [dNTPs] (low [dATP] (lane 2 of Figure 7(a)), low [TTP] (lane 3 of Figure 7(a)), low [dCTP] (lane 4 of Figure 7(a)), or low [dGTP] (lane 5 of Figure 7(a)) and the amount of DNA synthesized past the TNR insertion site at low [dGTP] (lane 5 of Figure 7(a)). The number of each dNTP that has to be incorporated up to the insertion site is also indicated underneath the panel of histograms. The same quantification as that described in 7(b) was performed with miniforks carrying a 5′GTT (c), 5′CAG (d) and 5′CTG (e) repeat. For the miniforks containing a TNR unit, R is the ratio between the amount of DNA synthesized past the TNR unit under a given condition of [dNTPs] (low [dATP], low [TTP], low [dCTP], or low [dGTP]) and the amount of DNA synthesized past the TNR unit at low [dGTP]. The number of each dNTP that has to be incorporated up to the end of the TNR unit is indicated underneath the panel of histograms.

At low concentration of dNTPs, the 5′CTG repeat located on the leading strand template created a greater impediment to the reconstituted gp41-gp43-gp59 replication complex than the 5′CAG repeat. To investigate which dNMP incorporation reaction was responsible for the impediment of the DNA polymerase, a coupled leading strand DNA synthesis assay was performed with one of the four dNTPs at a low concentration. The three other dNTPs were kept at a high concentration. We first established the DNA synthesis profiles of the gp41-gp43-gp59 replication trio across a ds random sequence. As shown in Figure 7(a), for a minifork containing a random sequence, the DNA synthesis profile was specific to each reaction condition. For instance, the reaction performed at low concentration of dATP (Figure 7(a), lane 2) gave shorter DNA synthesis products than the reaction performed at low concentration of dGTP (Figure 7(a), lane 5). To quantify this aspect of the reaction, we counted the number of each dNMP to be incorporated up to the TNR insertion site (indicated by a backward arrow in Figure 7(a)) and measured the amount of DNA that was synthesized past the TNR insertion site under each of the four reaction conditions (low [dATP], low [TTP], low [dCTP], or low [dGTP]). To facilitate comparisons of efficiency of DNA synthesis between the miniforks, the amount of DNA synthesized under a given condition was calculated relative to that formed under low concentration of dGTP. There was a clear inverse correlation between the number of dXMP to be incorporated up to the insertion site and the quantity of DNA that was synthesized past the TNR insertion site when this dXTP was present at a low concentration (Figure 7(b)). For instance, 20 dAMP and 9 dGMP had to be incorporated up to the insertion site, and the amount of DNA synthesized beyond the insertion site at low dATP concentration was 16 +/− 5% of that formed at low concentration of dGTP. The same inverse correlation applied to the minimal reconstituted T4 replisome synthesizing through the non structure-forming 5′GTT TNR sequence (Figure 7(c)). In the case of a minifork containing a given TNR sequence, the number of dXMP to be incorporated up to the end of the TNR unit and the amount of DNA synthesized beyond the TNR unit when this dXTP was present at a low concentration were compared. Surprisingly, this inverse correlation no longer held when the gp41-gp43-gp59 replication trio replicated through a structure-forming TNR (5′CAG or 5′CTG, Figures 7(d) and 7(e)). For instance, the incorporation of dGMP opposite dCMP of the 5′CAG or 5′CTG sequence gave the highest yield of DNA synthesis although the number of dGMP to be incorporated up to the end of the TNR unit (32 in case of the 5′CAG containing minifork and 27 in case of the 5′CTG containing minifork) was not the lowest among the four dNMP to be incorporated. In addition, for both miniforks containing a structure-forming TNR, the same amount of dAMP or TMP [37] had to be incorporated up to the end of the 5′CTG or 5′CAG unit, respectively (Figures 7(d) and 7(e)), but the relative amount of DNA synthesized past the 5′CAG unit under low concentration of TTP (40 +/− 13%) was roughly twice that synthesized past the 5′CTG unit under low concentration of dATP (22 +/− 6%) (Figures 7(d) and 7(e)). This result suggested that the incorporation of dAMP across TMP in the 5′CTG sequence context was a more difficult reaction than the incorporation of TMP across dAMP in the 5′CAG sequence context, thus giving an explanation for the greater impediment to the gp41-gp43-gp59 replication trio created by a 5′CTG unit than a 5′CAG unit at low dNTP concentrations.

## 4. Discussion

Repetitive sequences, including TNRs, are more prone to mutate than random sequences Ellegren [1]. Recently, we proposed a new model (called the template-push model) for the dependence of TNR instability on the orientation of the replication fork and the deletion bias observed *in vivo* for these repetitive sequences [26]. In this model, the TNR sequence that the replisome must replicate creates a greater hindrance for the progression of the leading than the lagging DNA polymerase; as a consequence, the replicative helicase and the leading DNA polymerase transiently uncouple their activities, and a short gap of ss DNA between the two proteins appears. To restore its coupling with the moving helicase and save time for DNA synthesis, the leading DNA polymerase passes over the small track of naked leading strand template without synthesizing DNA. By this mechanism, polymerase-helicase coupling is maintained but at the expense of a hairpin that is formed on the template strand after protein coupling has been re-established. If it is left unrepaired or is repaired in an error-prone manner, the hairpin can induce a deletion of the TNR unit at the next round of replication. In this paper, we describe a method to build replication miniforks suitable for testing the template-push model, and we show how the use of these miniforks brought insights into the mechanism of TNR instability.

Although very simple to prepare, the miniforks assembled from purchased oligonucleotides are limited in size by the length of the oligonucleotides that can be chemically synthesized (around 100 nts). Our method of preparation of replication miniforks overcomes this limitation since the production of ss leading and lagging strand templates relies on PCR followed by the specific degradation of one strand of the PCR product by the T7 exonuclease (Figure 1). As a consequence any sequence carried in a ds DNA can be used, making it possible to assemble a replication minifork of any given sequence. The Herculase-enhanced DNA polymerase and the Phusion high-fidelity DNA polymerase have both been successfully used to amplify sequences containing around 20 TNRs. The leading strand template indeed carries the expected number of repeats (see sequencing lanes in Figures 3, 4, 6, and 7). It is possible that the Herculase-enhanced DNA polymerase becomes more appropriate than the Phusion high-fidelity DNA polymerase when dealing with longer repeats because the former DNA polymerase can faithfully and efficiently cope with long G-C rich targets (Stratagene). 

The striking conservation of the DNA replication apparatus in bacteriophage T4 and in human cells [27] make the T4 DNA replication machinery an ideal simple model system to test *in vitro* the miniforks prepared by the method described above and investigate the activity of reconstituted replisomes of increasing complexities. A functional minimal replication complex composed of the T4 DNA polymerase and the T4 helicase can perform strand displacement DNA synthesis across a random sequence, nonstructure-forming and structure-forming TNRs (Figures 3 and 4). The T4 helicase loader stimulates both the DNA synthesis and the ATPase activities of the helicase-DNA polymerase replication couple, but interestingly to different extents. For instance, the twofold stimulation of the ATPase activity of the T4 helicase by the T4 helicase loader (Figure 5) is associated with the fivefold increase of leading strand DNA synthesis (Figure 4). As indicated by its name, the T4 helicase loader stimulates the loading of the T4 helicase around naked or SSB-covered ss DNA. Whether this factor dissociates after loading of the helicase is unclear, and it is possible that the T4 helicase loader remains part of the T4 replisome. If the T4 helicase loader travels with the replication complex and if most of the ATP is used to unwind the parental ds DNA (which we believe is true as under our experimental conditions, gp59 stimulates both the intrinsic and the ss-dependent ATPase activity of gp41), our result suggests that the T4 helicase loader prevents the slippage of T4 helicase, by reducing the amount of ATP hydrolyzed that is not associated with forward translocation. 

Using the method that quantifies the extent of impediment created by various TNR sequences to the progression of DNA polymerases [26] and by applying it to minimal reconstituted T4 replication complexes, we found that at high concentrations of dNTPs a 5′CAG leading strand template creates a greater impediment to the gp41-gp43-gp59 replication trio than a 5′CTG leading strand template (Figure 4). A similar ranking of these two sequences was reported when naked 5′CAG and 5′CTG sequences were tested in a primer extension assay that did not required the T4 helicase [26]. This result suggests that the ss DNA exposed either by the T4 helicase or by a chemical denaturing treatment is similarly converted into ds DNA by the T4 DNA polymerase. In contrast, when the concentrations of all dNTPs are low, a 5′CTG leading strand sequence more dramatically hinders the progression of the gp41-gp43-gp59 replication trio than a 5′CAG leading strand sequence (Figure 6), suggesting a change in the rate-limiting step when the concentration of dNTPs varies. It is possible that at low dNTP concentrations, the binding of the incoming complementary dNTP by the DNA polymerase becomes the rate-limiting step of the coupled DNA synthesis reaction, whereas at high dNTP concentrations another step, such as the chemical incorporation of dNMP or the translocation of the DNA polymerase after the dNMP incorporation, becomes rate limiting. 

By keeping a single (out of the four) dNTPs at a low concentration, it was found that the incorporation of dAMP across TMP of the 5′CTG sequence is a more difficult reaction than the incorporation of TMP across dAMP of the 5′CAG sequence context (Figure 7), thus giving an explanation for the greater hindrance to progression of the minimal reconstituted T4 replisome created by a 5′CTG sequence than a 5′CAG sequence under low concentration of dNTPs (Figure 6). It is well known that dNTPs are not all four present at the same concentration in the cell, TTP being the most abundant dNTP [28–30], and that specific imbalanced pools of dNTPs can be mutagenic [31]. In *E. coli*, the pool of dNTPs drops during cell growth and when the cell transits from exponential growth to stationary phase [29]. Similarly, confluent human cells have a low pool of dNTPs [30]. In addition, in eukaryotic cells, the pool of dNTPs is tightly regulated during the cell cycle and peaks during the S, G2 and M phases [28, 31–33]. During the G1 phase, the amount of dNTPs is low and the use of dNTPs is restricted to mitochondrial DNA synthesis and repair. The fact that the concentration of dNTPs can regulate the progression of the gp41-gp43-gp59 replication trio across a 5′CAG/5′CTG TNR unit in a strand-dependent manner suggests that the repair of a lesion or a gap located in a 5′CAG/5′CTG sequence context, can have different outcomes if it takes place in the G1 or outside the G1 phase of the cell cycle. For instance, during base excision repair, after removal of a 2-hydroxyadenine located in a 5′CAG sequence context, dAMP needs to be incorporated opposite TMP of the CTG repeat. The one-nt gap intermediate formed after the excision of the lesion may have more chance to slip and to give rise to frameshift mutations during the G1 phase than early in the S phase before the passage of the replisome or during the G2 phase. At this stage of the cell cycle, the pool of dNTPs is indeed low and, as pointed out by our experiments, incorporation of dAMP across TMP is difficult. Our results therefore point to a specific role of the phase of the cell cycle (G1 *versus* G2 or S) and the state of the cell (dividing *versus* nondividing) at which DNA lesions are repaired. 

## Figures and Tables

**Table 1:** Description of plasmids and oligonucleotides.

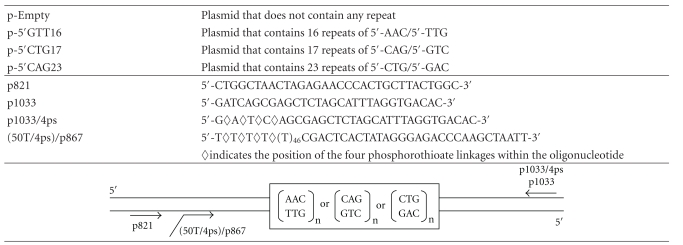

The upper part of the table describes the plasmids. The middle part of the table refers to the oligonucleotides and their sequences. The lower part of the table shows the hybridization positions of the oligonucleotides.

## Abbreviations

ATP: Adenosine triphosphate
bp: Base pair
ds: Double-stranded
dNTP: Deoxynucleoside triphosphate
dNMP: Deoxynucleoside monophosphate
DTT: Dithiothreitol
EDTA: Ethylenediaminetetraacetic acid
EtBr: Ethidium bromide
gp: gene product
nt: Nucleotide
PCR: Polymerase chain reaction
PEI: Polyethylene imine
PNK: Polynucleotide kinase
p-t: Primer-template
1X TBE: 90 mM Tris, 90 mM Borate and 2 mM EDTA
TE: 10 mM Tris-HCl pH 7.8 and 1 mM EDTA
TLC: Thin layer chromatography
TNR: Trinucleotide repeat
ss: Single-stranded
SSB: Single-stranded binding.
